# Serological evaluation for the current epidemic situation of foot and mouth disease among cattle and buffaloes in Egypt

**DOI:** 10.14202/vetworld.2020.1-9

**Published:** 2020-01-03

**Authors:** Mariam M. Abd El-Rhman, Diea G. Abo El-Hassan, Walid S. Awad, Sayed A. H. Salem

**Affiliations:** 1 Department of Preventive Medicine, General Organization for Veterinary Services, Dokki, Giza, Egypt; 2 Department of Internal Medicine and Infectious Diseases, Faculty of Veterinary Medicine, Cairo University, Giza, Egypt; 3 Department of Virology, Animal Health Research Institute, Dokki, Giza, Egypt

**Keywords:** 3ABC enzyme-linked immunosorbent assay test, Egypt, foot-and-mouth disease virus, non-structural protein, solid-phase competitive enzyme-linked immunosorbent assay

## Abstract

**Aim:**

The present study was aimed to investigate the epidemic situation of foot-and-mouth disease (FMD) in Egypt from 2016 to 2018 based on the detection of FMD virus (FMDV) in carrier or previously infected animals, by determination of antibodies against non-structural protein (NSP), implementation a pilot study on circulating FMDV serotypes and assure the efficacy of locally produced inactivated trivalent vaccine.

**Materials and Methods:**

A total of 1500 sera were collected from apparent healthy vaccinated cattle and buffaloes from three Egyptian geographical sectors, representing ten governorates. Determination of FMD antibodies against NSP was carried out using 3ABC enzyme-linked immunosorbent assay (ELISA) test. Serotyping of the circulating FMDV and assure the vaccine efficacy was performed using solid-phase competitive ELISA.

**Results:**

The 3ABC ELISA test revealed 26.4% and 23.7% positive for FMDV-NSP antibodies in cattle and buffalo sera, respectively. The highest positivity was in Delta Sector among both cattle 42.3% and buffaloes 28.8%. Serotyping of FMDV-positive NSP sera in El-Qalyubia Governorate for the circulating FMDV serotypes O, A, and Southern African Territories (SAT) 2 was 52.2%, 17.4%, and 30.4% in cattle and 31.8%, 27.3%, and 40.9% in buffaloes, respectively. The overall protection level due to the vaccination program was 62.1 and 60.9% in cattle and buffaloes, respectively, while the protective level of the FMDV serotypes O, A, and SAT2 included in the inactivated trivalent vaccine was 73.9, 84.6, and 63.8% in cattle and 72.3, 82.3, and 63.5% in buffaloes, respectively.

**Conclusion:**

The present study recommended full determination for the immunogenic relationship between the vaccine strains and the field strains to attain maximum protection against the circulating viruses.

## Introduction

Foot-and-mouth disease (FMD) is one of the most important contagious viral transboundary animal diseases affecting cloven-hoofed animals, including cattle, buffaloes, pigs, sheep, and goats [1], characterized by vesicular lesions in the mouth and on the feet, teats, and nares, causing low mortality rate in adult animals, but often high mortality in young due to myocarditis [2], with important economic losses and restriction of international animal trade [3]. The etiological agent, FMD virus (FMDV), a single-stranded positive-sense RNA is classified within the genus *Aphthovirus* in the family *Picornaviridae* [4]. The virus exists in seven serologically and genetically distinguishable serotypes: O, A, C, Southern African Territories (SAT), 1-3, and Asia-1 [5]. Several of these serotypes are circulating currently or periodically in the Middle East and North Africa [6]. Within each serotype, there are several topotypes (genetically and geographically) which are further divided into various genotypes (lineages or strains), with up to 61 described so far, as a result of mutation from error-prone RNA replication, recombination, and host selection [7]. The emergence of new serotypes or topotypes has been associated with the importation of animals from endemic countries and the use of incompletely matching vaccines, which made the animals prone to infections with antigenically atypical strains of FMDV [8]. The viral RNA is translated into a single polypeptide which is then cleaved by viral proteases into 12 viral proteins, classified into four structural proteins (SPs) (VP1, VP2, VP3, and VP4) that form the icosahedral viral capsid, and eight non-structural proteins (NSPs) (L, 2A, 2B, 2C, 3A, 3B, 3C, and 3D) that participate in viral replication and play other functions within the host cell; and during the cleavage, the 3A, 3B, 3C, or 3A, 3B proteins are also combined to form 3ABC or 3AB protein complex [9]. SPs are more variable than NSPs and mutations or deletions in SPs help FMDV to evade an immune response produced by the host [10].

FMD is enzootic in Egypt since 1950s, it remains a serious threat to cattle and buffaloes population [11,12]. Serotype O has a long history in Egypt with many topotypes and lineages, serotype A was reported in 2006 followed by SAT2 serotype in 2012 [13-15]. The prevention strategy to avoid FMD outbreaks occurrence in Egypt is vaccination using locally produced (O Panasia-2/A Iran-05/SAT2/EGY-A-2012) trivalent inactivated vaccine [16]. Vaccination is a major tool for FMD control to mitigate the impact of clinical disease, or to reduce and eventually eliminate virus circulation as outlined in the Progressive Control Pathway for FMD control [17]. From 2012 to 2018, FMD outbreaks have struck cattle and buffaloes in different localities of Egypt exerting severe economic losses to livestock industries. As vaccine strains used in a particular geographical region mainly depend on the serotypes and genotypes circulating in the region and FMD SP antibody levels are strongly correlated with protection, population immunity is estimated through field surveys for NSPs and the protective antibody levels as part of routine post-vaccination monitoring to identify areas with low protection [18-20]. Enzyme-linked immunosorbent assays (ELISAs) used to detect FMD viral antigens as well detection and serotyping of specific antibodies to SPs using inactivated or recombinant antigens [21]. Solid-phase competitive ELISA (SPCE) has replaced complement fixation in most laboratories as it is more specific and sensitive [22]. No significant difference is usually observed between the VNT and SPCE for both screening and titration of FMDV serotypes. Moreover, SPCE has the advantage that the test is rapid (the result can be read in 1 day vs. waiting 3 days in VNT) and easier to perform [23]. Detection of antibodies to NSPs of FMDV is useful in providing evidence of previous or current viral replication in the host, irrespective of vaccination status. A recombinant FMDV NSP of 3ABC, an indirect ELISA, was established to specifically identify antibodies induced by FMDV infection but not those induced by vaccination [24].

The present work aimed to clarify the current serological situation of FMD in cattle and buffaloes in Egypt through a cross-sectional field study. The study will assess the post-vaccination NSPs and SP antibody levels in a cohort of vaccinated cattle and buffaloes will be vaccinated within the Egyptian FMD vaccination program, to define the circulating FMDV strains and evaluate vaccine protection in the population at large.

## Materials and Methods

### Ethical approval

This study protocol was approved by the Institutional Animal Care and Use Committee, the Ethics Committee of the Faculty of Veterinary Medicine, Cairo University, Giza, Egypt (Approval No. VetCU0722019060).

### Study area and period of study

**Figure-1:**
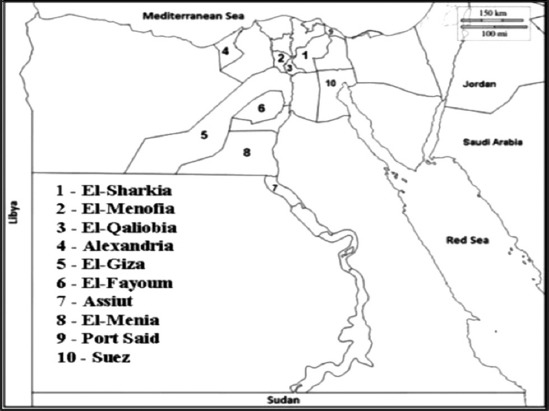
Ten Egyptian Governorates included in the study (area of study).

Three Egyptian sectors including ten governorates (Figure-1) were included in the study; these sectors are Delta (El-Sharkia, El-Menofia, El-Qalyubia, and Alexandria), Upper Egypt (El-Giza, El-Fayoum, Assiut and El-Menia), and Suez Canal (Port Said and Suez) during the period between December 2016 and December 2018.

### Animals and samples

A total of 1500 whole blood samples without anticoagulant were collected from clinically examined, apparently healthy vaccinated 739 cattle and 761 buffalo, 4 weeks post-vaccination. Blood samples were collected from jugular vein on serum separator tubes, clear sera were collected and stored at −70°C till tested. Cattle and buffalo were vaccinated by veterinary authorities using oil-adjuvant trivalent vaccine containing (O Pan Asia2, A Iran O5, and SAT2/EGY/2012 FMDV serotypes; payload of antigens of 6.5, 6.2, and 5.9, μg/dose/from each serotype, respectively) formulated in a double oil emulsion adjuvant produced by Veterinary Serum and Vaccine Research Institute, Cairo, Egypt.

### Differentiation of infected and vaccinated animals (DIVA test)

Performed on the basis of the detection of antibodies against NSPs of FMDV in sera of carrier or previously infected animals and their absence in that of non-infected and vaccinated animals. Detection of antibodies against NSPs was done using prioCHECK^®^ 3 ABC NSP ELISA kit (Prionics AG, Zurich, Switzerland). The test uses anti-3ABC specific monoclonal antibodies (MAbs) coated to the solid phase to trap the recombinant 3ABC polypeptide expressed in *E. coli*. Test sensitivity and specificity are 98% and 96%, respectively.

### Serotyping of FMDV in positive NSPs antibodies

A total of 45 positive serum samples for antibodies against NSPs of FMDV (23 and 22 out of 40 and 60 cattle and buffalo from El-Qalyubia Governorate, a member of Delta Sector) were serotyped through detection of antibodies specific to serotypes O, A, and SAT2 of FMDV using SPCE (IZSLER^®^ Biotech laboratory, Brescia, Italy). The assay was done using neutralizing anti-FMDV MAbs specific for FMDV serotypes O, A, and SAT2 to measure antibodies against these serotypes in serum samples of FMDV previously or currently replication in the host, irrespective of vaccination status. ELISA microplate was supplied pre-coated with the FMDV types O, A, and SAT2 inactivated antigen captured by the homologous MAb. Test sensitivity and specificity were 94% and 92%, respectively. The results of the screened sera were calculated according to its inhibition percentage, according to the manufacturer’s test guidelines.

### Detection of protection level of FMD vaccine (post-vaccination serosurveillance) in negative NSPs serum samples

Negative serum samples for NSPs antibodies against FMDV were subjected to SPCE (IZSLER^®^ Biotech laboratory, Brescia, Italy) for detection of the antibodies titer specific to serotypes O, A, and SAT2 (semi-quantitative). The measured antibody titer was classified according to the reference laboratory of Animal Health Research Institute, Giza, Egypt into: Negative, not protective, and protective.

### Sample analysis

On the basis of the detection of antibodies against NSPs of FMDV in sera of infected animals, the results were calculated according to the manufacturer’s test guidelines. Test sera are considered positive when producing inhibition of ≥50% and negative when producing an inhibition <50%.

On the basis of the circulating FMDV serotypes recorded in Egypt; O, A, and SAT2 and consequently vaccination performed using inactivated trivalent vaccine include these serotypes; the overall protective levels of the vaccine expected to protect animals against the recorded circulating FMDV serotypes are classified into: Negative (at least antibodies titer of one serotype is negative), not protective (antibodies titer against all the three serotypes is not protective or at most antibodies titer of only two serotypes is protective), and protective (antibodies titer against all the three serotypes is protective).

On the basis of an evaluation of the protective antibodies levels of single serotype included in the trivalent vaccine, each serotype antibody titer was calculated semi-quantitatively according to the manufacturer’s test guidelines. Test sera were considered negative when producing an inhibition <70% at the 1/10 dilution and the second dilution (1/30) provides an indication of the level of antibodies. Low positive sera (not protective level) show ≥80% inhibition at the 1/10 dilution but ≤50% inhibition at the 1/30 dilution, while the strong positive sera (protective level) show ≥80% inhibition at both 1/10 and 1/30 dilutions.

### Statistical analysis

The statistical analysis of data was done by the Chi-square test using SPSS v20. The results were considered significantly different with p<0.05.

## Results

### DIVA test

A total of 375 sera out of 1500 vaccinated cattle and buffalo were positive for antibodies against NSPs of FMDV by DIVA test using 3ABC ELISA. Out of the 375 positive sera, 195/739 cattle (26.4%) and 180/761 buffalo (23.7%) were detected. The prevalence of previous or current infection among cattle in the selected geographical sectors; Delta, Upper Egypt, and canal were 42.3, 15.2, and 17.1% while among buffalo was 28.8, 14.3, and 10.0%, respectively, as shown in Table-1.

**Table-1:** Detection of antibodies against NSPs of FMDV (DIVA test) in cattle and Buffaloes sera using 3ABC ELISA.

Sectors	NSPs in cattle sera			NSPs in buffalo sera		
	
	Examined (n)	Positive (%)	Negative (%)	Examined (n)	Positive (%)	Negative (%)
Delta	300	127 (42.3)	173 (57.7)	500	144 (28.8)	356 (71.2)
Upper Egypt	369	56 (15.2)	313 (84.8)	231	33 (14.3)	198 (85.7)
Canal	70	12 (17.1)	58 (82.9)	30	3 (10.0)	27 (90.0)
Total	739	195 (26.4)	544 (73.6)	761	180 (23.7)	581 (76.3)

NSPs=Non-structureal protein, ELISA=Enzyme-linked immunosorbent assay, FMD=Foot-and-mouth disease virus

Out of 1125 negative sera, 544/739 (73.6%) from cattle and 581/761 (76.3%) from buffalo were detected. Antibody prevalence due to vaccination among cattle in the selected geographical sectors; Delta, Upper Egypt, and canal were 57.7, 84.8, and 82.9% while among buffalo was 71.2, 85.7, and 90.0%, respectively, as shown in Table-1.

### Serotyping of FMDV in positive NSPs antibodies

A total of 23/40 (57.5%) and 22/60 (36.7%) sera of vaccinated cattle and buffalo from El-Qalyubia Governorate, a member of Delta Sector, were positive for FMDV NSPs antibodies using 3ABC ELISA, respectively. Screening of that sera for antibodies against FMDV serotypes O, A and SAT-2 using SPC ELISA test revealed 12 (52.2%), 4 (17.4%), and 7 (30.4%) in cattle sera and 7 (31.8%), 6 (27.3%) and 9 (40.9%) in buffalo sera, respectively. Serotype “O” was the pre-dominant serotype in cattle while serotype “SAT-2” was the pre-dominant serotype in buffaloes in this geographical sector.

### Protection level of FMD vaccine in negative NSPs serum samples

Negative NSPs of FMDV sera were examined using SPCE to measure antibody titer of FMDV serotypes O, A, and SAT2 to detect the protective level of the locally used trivalent inactivated vaccine (Table-2). Results of the overall vaccine protective level were classified into three levels as negative antibody titer, not protected antibody titer, and protected antibody titer. The results in cattle were 42 (7.7%), 164 (30.2%), and 338 (62.1%), respectively, whereas in buffaloes were 60 (10.3%), 167 (28.7%), and 354 (60.9%), respectively. According to the geographical sectors (Delta, Upper Egypt, and Canal), the protective levels in cattle were 20 (11.6%), 50 (28.9%), and 103 (59.5%); 19 (6.1%), 99 (31.6%), and 195 (62.3%), and 3 (5.2%), 15 (25.9%), and 40 (68.9%), respectively, whereas in buffaloes were 36 (10.1%), 102 (28.7%), and 218 (61.2%); 23 (11.6%), 58 (29.3%), and 117 (59.1%), and 1 (3.7%), 7 (25.9%), and 19 (70.4%), respectively, as shown in Table-2.

**Table-2:** Overall protection level of FMD vaccine (post-vaccination serosurveillance) in negative NSPs serum samples in cattle and buffaloes using SPCE.

Overall vaccine protection level	Cattle no. (%)/Sector			Total cattle no. (%) (544)	Buffalo no. (%)/Sector			Total buffalo no. (%) (581)
	
	Delta (173)	Upper Egypt (313)	Canal (58)		Delta (356)	Upper Egypt (198)	Canal (27)	
Negative	20 (11.6)	19 (6.1)	3 (5.2)	42 (7.7)	36 (10.1)	23 (11.6)	1 (3.7)	60 (10.3)
Not protective	50 (28.9)	99 (31.6)	15 (25.9)	164 (30.2)	102 (28.7)	58 (29.3)	7 (25.9)	167 (28.7)
Protective	103 (59.5)	195 (62.3)	40 (68.9)	338 (62.1)	218 (61.2)	117 (59.1)	19 (70.4)	354 (60.9)

Negative=At least antibodies titer of one serotype is negative, Not protective=Antibodies titer against all the three serotypes is not protective or at most antibodies titer of only two serotypes is protective, Protective=Antibodies titer against all the three serotypes is protective, NSPs=Non-structureal protein, SPCE=Solid-phase competitive ELISA, FMD=Foot-and-mouth disease

The measurement of protection level of each single serotype (O, A, and SAT2) included in the local inactivated trivalent FMD vaccine in the negative NSPs sera using SPCE (Table-3) was classified into negative antibody titer, not protective antibody titer, and protective antibody titer. The results in the vaccinated cattle were 21 (3.9%), 121 (22.2%), and 402 (73.9%); 13 (2.4%), 71 (13.1%), and 460 (84.6%), and 38 (7.0%), 159 (29.2%), and 347 (63.8%), respectively, whereas in the vaccinated buffaloes were 35 (6.0%), 126 (21.7%), and 420 (72.3%); 14 (2.4%), 89 (15.3%), and 478 (82.3%); and 49 (8.4%), 163 (28.1%), and 369 (63.5%), respectively.

**Table-3:** Protective level of serotypes (O, A, and SAT2) included in trivalent FMD vaccine in negative NSP serum samples of cattle and buffaloes using SPCE.

Serotype	Protective titer	Cattle no. (%)/Sector			Total cattle no. (%) (544)	Buffalo no. (%)/Sector			Total buffalo no. (%) (581)
	
		Delta (173)	Upper Egypt (313)	Canal (58)		Delta (356)	Upper Egypt (198)	Canal (27)	
O	N	11 (6.4)	8 (2.6)	2 (3.5)	21 (3.9)	22 (6.2)	13 (6.6)	0 (0.0)	35 (6.0)
	NP	41 (23.7)	69 (22.0)	11 (19.0)	121 (22.2)	71 (19.9)	51 (25.8)	4 (14.8)	126 (21.7)
	P	121 (69.9)	236 (75.4)	45 (77.6)	402 (73.9)	263 (73.9)	134 (67.7)	23 (85.2)	420 (72.3)
A	N	8 (4.6)	5 (1.6)	0 (0.0)	13 (2.4)	9 (2.5)	5 (2.5)	0 (0.0)	14 (2.4)
	NP	20 (11.6)	45 (14.4)	6 (10.3)	71 (13.1)	62 (17.4)	27 (13.6)	0 (0.0)	89 (15.3)
	P	145 (83.8)	263 (84.0)	52 (89.7)	460 (84.6)	285 (80.1)	166 (83.8)	27 (100.0)	478 (82.3)
SAT2	N	17 (9.8)	18 (5.8)	3 (5.2)	38 (7.0)	30 (8.4)	18 (9.1)	1 (3.7)	49 (8.4)
	NP	53 (30.6)	92 (29.3)	14 (24.1)	159 (29.2)	93 (26.1)	63 (31.8)	7 (25.9)	163 (28.1)
	P	103 (59.5)	203 (64.9)	41 (70.7)	347 (63.8)	233 (65.5)	117 (59.1)	19 (70.4)	369 (63.5)

N=Negative antibody titer, NP=Not protective antibody titer, P=Protective antibody titer, NSP=Non-structureal protein, FMD=Foot-and-mouth disease, SPCE=Solid-phase competitive ELISA

According to the geographical sectors, the protective levels of serotypes (O, A and SAT-2) were 11 (6.4%), 41 (23.7%) and 121 (69.9%); 8 (4.6%), 20 (11.6%) and 145 (83.8%); 17 (9.8%), 53 (30.6%) and 103(59.5%) among cattle, while buffaloes revealed 22 (6.2%), 71 (19.9%) and 263 (73.9%); 9 (2.5%), 62 (17.4%) and 285 (80.1%); 30 (8.4%), 93 (26.1%) and 233 (65.5%) in Delta sector respectively. In Upper Egypt sector, cattle demonstrated 8(2.6%), 69 (22.0%) and 236 (75.4%); 5 (1.6%), 45 (14.4%) and 263 (84.0%); 18 (5.8%), 92 (29.3%) and 203 (64.9%) while buffaloes showed 13 (6.6%), 51 (25.7%) and 134 (67.7%); 5 (2.5%), 27 (13.7%) and 166 (83.8%); 18 (9.1%), 63 (31.8%) and 117 (59.1%) respectively. In Canal sector, cattle demonstrated 2 (3.4%), 11 (19.0%) and 45 (77.6%); 0 (0.0%), 6 (10.3%) and 52 (89.7%); 3 (5.2%), 14 (24.1%) and 41 (70.7%) while buffaloes revealed 0 (0.0%), 4 (14.8%) and 23 (85.2%); 0 (0.0%), 0 (0.0%) and 27 (100.0%); 1 (3.7%), 7 (25.9%) and 19 (70.4%) respectively.

## Discussion

FMD remains a serious threat to millions of cattle and buffaloes population around the globe and remains the main sanitary barrier to the commerce of animals and animal products [18,25]. Egypt is an endemic country with three FMDV serotypes, namely, O, A, and SAT2 resulting in negative impacts for farmers including direct production losses and indirect losses related to implementing control measures [14,18,26,27]. Despite systematic use of vaccination, severe yearly outbreaks of FMD have been recorded in different localities of Egypt, and there is evidence of sustained virus circulation in vaccinated cattle and buffalo populations [16,28-31], cattle and buffaloes are considered the main dairy animals at risk to FMD [32]. The present work was carried out through a cross-sectional field study included ten Egyptian Governorates during 2016-2018. We assessed post-vaccination antibody levels against NSPs and SP of FMDV, to gather evidence with respect to the FMDV circulation and vaccine protection in the population at large. Antibodies against NSPs of FMDV were detected in 26.4% of cattle sera and 23.7% of buffaloes sera (Table-1), with no significant statistical difference (p=0.530), revealing previous or current infection with FMDV among cattle and buffaloes in the area of study. These results were similar to that obtained by Abd El-Rhman *et al*. [11] who recorded NSPs of FMDV in 27.6% of cattle and 22.0% of buffaloes with non-significant species difference (p=0.78); Raof *et al*. [27] who recorded 27.1% for cattle and 35.7% for buffaloes; Ateya *et al*. [33] who recorded 15.5% in vaccinated cattle and not agree with Mesfine *et al*. [34]; and El Bahgy and Moustafa [35] who reported high statistically significant difference (p<0.05) between cattle and buffaloes in NSPs of FMDV seroprevalences. Of note, antibodies against NSPs of FMDV were detected in the three selected Egyptian geographical sectors; Delta was the highest sector 42.3% and 28.8%, for cattle and buffaloes, respectively. The prevalence of infection in the different geographical sectors ranged from 15.2% to 42.3% among cattle, whereas it ranged from 10% to 28.8% among buffaloes (Table-1); this result is nearly similar with that of Kamel [36] and Shabana [37] where the average prevalence of FMD infection in vaccinated bovine population ranged from 8% to 32%. In statistical terms, there is a statistically significant difference in NSPs seroprevalence of FMD in Delta Sector (p=0.024) as well as between cattle and buffaloes in the same sector (p=0.043); this significance coincides with Ahmed *et al*. [29] who reported that Delta Governorates characterized by its high density of animals’ population, so early struck of infection firstly was recorded in Delta region as in the new outbreak of FMDV serotype SAT2. These results supported by Paton *et al*. [38] who reported that NSP 3ABC antibody is an accurate monitoring method for virus circulation and reliable measurement of the antibody status of infected and vaccinated animals.

In developing countries including Egypt where FMD is endemic, serological assays and serotyping of field strains can give a precise and more accurate picture of the disease topography [39]. Our results of serotyping of FMD on positive NSPs cattle and buffalo sera, belong to El-Qalyubia governorate in Delta Sector, using SPCE, revealed the prevalence of serotypes O, A, and SAT2 among cattle in percentages of 52.2, 17.4, and 30.4%, respectively, whereas among buffaloes in percentages of 31.8%, 27.3%, and 40.9%, respectively. These results give an indication of the circulating serotypes among cattle and buffaloes, in a pilot study was carried out in the highest disease prevalence sector. These results are in agreement with the data of Egyptian FMDV serotypes recorded by Diab *et al*. [2] who detected serotype O, A, and SAT2 among cattle, and FAO [40] which published that FMDV serotypes O, A, and SAT 2 were reported to OIE in 2012-2017 from cattle and buffaloes in Egypt. Cattle were significantly appeared to be more infected with serotype “O”, 52.2% (p=0.022), followed by “SAT2,” 30.4% (p=0.037), and “A,” 17.4% (p=0.975). Otherwise buffaloes were significantly appeared to be more infected with serotype “SAT2,” 40.9% (p=0.024), followed by “O,” 31.8% (p=0.041), and “A,” 27.3% (p=0.749). These results are in agreement with Abd El-Rhman *et al*. [11] and Kandeil *et al*. [41] who reported that serotype “O” was highly detected in field samples and predominantly circulating among cattle and buffaloes in most of the Egyptian Governorates. Serotype “O” causes more than 60% of the FMD outbreaks worldwide, especially in East Africa and Medill east [6,9,42]. The obtained results agreed with Elhaig and Elsheery [43] who mentioned that buffaloes appeared to be more susceptible to the “SAT2” than cattle. Whereas buffaloes FMDV strains genetically distinct from the strains obtained from cattle [44], the present study strengthens FMD surveillance in buffaloes as they could pose a potential risk of virus transmission to cattle. From a broader perspective, despite the strict control policies and quarantine measures at the borders, similar results to NSPs antibodies of FMDV that obtained in Egypt were recorded in Libya (19.0% [45], Sudan (25.0%)[46], Ethiopia (24.2%) [47], Saudi Arabia (25.0%) [48], Oman (26.8%) [49], and India (27.7%) [21], indicating a risk of transboundary transmission of new topotypes/lineages of the virus. Regional cooperation including data/information and applied control measures on the FMD outbreaks is required [50].

In the present work, negative NSPs of FMDV sera were examined using SPCE to measure the overall protective level of the locally used trivalent inactivated vaccine as well as the antibody titer of FMDV serotypes O, A, and SAT2 included. It is supported by Ehizibolo *et al*. [51] who reported that SPCE has been validated for evaluation antibody titer in cattle and buffaloes sera. Results of the overall vaccine protective level were classified into three levels as negative, susceptible to infection, not protective, animals under risk, and protective; in cattle, the level percentages were 7.7%, 30.2%, and 62.1%, respectively, whereas in buffaloes were 10.3, 28.7, and 60.9%, respectively (Table-2); with no statistically significant difference in protective levels between cattle and buffaloes (p=0.994). Buffaloes were more susceptible to infection than cattle (p=0.793), while cattle were more under risk to infection than buffaloes (p=0.690). These results are in agreement with Abd El-Rhman *et al*. [11] who recorded protected levels 60.77% among vaccinated cattle and 76.65% among vaccinated buffaloes, in five Egyptian Governorates during the period 2013-2015; with no statistical significance difference in protective levels between cattle and buffaloes. Current decrease in the protection level among buffaloes may be due to the susceptibility of buffaloes to two distinct sublineages of SAT2 circulating in Egypt [2,29]. On the other hand, Shabana [37] reported vaccination program in Egypt could protect about 63% of animals, 20% of animals under risk, and 17% of animals still susceptible to infection; this may be due to FMDV serotype SAT2 was officially reported in Egypt in 2012 and not detected before [28]. According to geographical sectors, vaccination of cattle and buffaloes in Canal Sector showed the highest protective level, 68.97 and 70.37%, respectively. This may be due to the low animal density in this sector.

The measurement of protection level of each single serotype (O, A, and SAT2) included in the locally produced trivalent FMD vaccine, in negative NSPs sera using SPCE, was classified into negative, not protective, and protective (Table-3). The obtained results of serotype “O” in cattle revealed 3.9%, 22.2% and 73.9% while buffaloes revealed 6%, 21.7% and 72.3% for negative, not protective and protective respectively. Serotype”A” in cattle revealed 2.4%, 13.1% and 84.5% while buffaloes revealed 2.4%, 15.3% and 82.3% for negative, not protective and protective respectively. Serotype “SAT-2” in cattle revealed 7.0%, 29.2% and 63.8% while buffaloes revealed 8.4%, 28.1% and 63.5% for negative, not protective and protective respectively. Vaccinated cattle and buffaloes under screening showed susceptibility as well as high risk to infection with FMDV serotypes “SAT2” and “O,” respectively, especially in buffaloes. These results are in agreement with Elhaig and Elsheery [43] who mentioned that buffaloes appeared to be more susceptible to “SAT2” than cattle and Abd El-Rhman *et al*. [11] who recorded protective antibody titer of 69.67, 70.90% for serotype “O;” 79.88, 73.99% for serotype “A;” and 59.16, 58.20% for serotype “SAT2” in vaccinated cattle and buffaloes, respectively, in five Egyptian Governorates during the period 2013-2015. A slight improvement was detected in the population immunity, but still less than the required level of protection. According to geographical distribution, Canal Sector showed the highest protective level 77.6, 85.2% for serotype “O;” 89.7, 100.0% for serotype “A;” and 70.7, 70.4% for serotype “SAT2” in vaccinated cattle and buffaloes, respectively. Overall, high protective level was detected among the vaccinated cattle in Upper Egypt sector, while among the vaccinated buffaloes high protective level was detected in Delta Sector (Table-3). This may be due to high density of buffaloes in Delta than Upper Egypt.

In endemic countries, culling is not usually considered a viable control option due to the associated costs and stakeholder resistance. Therefore, FMD is generally controlled through vaccination [52], vaccination is the cheapest and effective method of disease control and limiting the spread of FMD [6,25]. Regular vaccination of cattle and buffalo against FMD in Egypt, has become an important input to maintain animal productivity and to reduce economic losses [20], but not prevent carrier state [22,53]. The current vaccine is inactivated trivalent FMD vaccine of serotypes (O Pan Asia2, A Iran O5, and SAT2/EGY/2012) [2,41]; of antigenic mass 6.5, 6.2, and 5.9, respectively [18]. To induce the permissible protection in vaccinated livestock, the antigenic mass of this vaccine should not be <2.2 μg/dose/from each serotype [54]. Although farmers and governments dedicate large amounts of resources to purchasing and administering FMD vaccines [16], many outbreaks of FMD have been reported even after vaccination [55]. This was observed in our finding and it coincides with Jamal *et al*. [56] who reported low humoral immune responses (low protective antibody titer) against FMDV by the locally produced vaccines. The protection conferred by the vaccine is usually serotype-specific and sometimes incomplete within a serotype [2,57], this affects the application of vaccine in the field [6,53,58], and may be result in vaccination failure due to differences between the circulating field strains and the strains included in the vaccine [3,59]. This agrees with Diab *et al*. [2] and Ahmed *et al*. [29] who performed phylogenetic analysis for FMD strains isolated from cattle in Egypt revealed that serotype O (EA-3) topotype was predominant but showed 14.6% differences from vaccine strain (O/PanAsia-2) of ME-SA topotype, Egyptian SAT2 virus was clustered into two distinct sublineages (ALX and GHB) within topotype VII as well as Valdazo-González *et al*. [15] who stated that FMDV diversity is high among SAT serotypes, especially for the SAT2, which is composed of 14 topotypes. Based on these effectiveness estimates, vaccination alone is unlikely to produce the high levels of herd immunity needed to control FMD, in cattle and buffaloes, without additional control measures [52].

## Conclusion

The present study highlights the recent serological situation of FMD in Egypt and it indicated that the application of vaccination program in governorates needs to be improved. Further work is warranted to ascertain the suitability of the existing serotype O and SAT2 vaccines to protect from the currently circulating viruses, and to establish if there is a need to develop a new vaccine. As vaccination does not prevent carrier state especially in buffalo’s population, vaccination alone is unlikely to control the disease unless it is coupled with animal movement control and animal identification systems.

## Authors’ Contributions

MMA, DGA, WSA, and SAHS contributed equally to the design of the work. SAHS, MMA, and DGA contributed equally to performing the work. MMA, DGA, and WSA contributed to data analysis. MMA contributed to sample collection. DGA, MMA, and WSA contributed to writing the work. All authors read and approved the final manuscript.
